# POSSIBLE SELVES THROUGH CAREGIVING: CAREGIVING HISTORY'S EFFECTS ON SELF-PERCEPTIONS OF AGING

**DOI:** 10.1093/geroni/igac059.378

**Published:** 2022-12-20

**Authors:** Rita Hu, Marina Larkina

**Affiliations:** University of Michigan, Ann Arbor, Michigan, United States; University of Michigan, Ann Arbor, Michigan, United States

## Abstract

Most studies examine the consequences of self-perceptions of aging (SPA). Little is known about the antecedents. The possible selves framework suggests that earlier life experiences can shape people’s scenarios about their own aging. We examine the association between early-life informal caregiving experiences and SPA later in life using data from the HRS Retrospective Life History Mail Survey and the Psychosocial and Lifestyle Questionnaire ( N = 2,556, Mage = 72.9). Participants reported up to five periods of unpaid caregiving (≥ 6 months) with family members. SPA was measured by the HRS 8-item scale. Step-wise linear regression revealed that compared with people who had not been caregivers, early-life caregivers reported more negative SPA later in life (β = 0.20, 95%CI[0.12, 0.29]). The association holds after controlling for health-related and demographic covariates. Future research and interventions should focus on the consequences of early-life caregiving on the caregiver’s scenarios about their own aging.

